# Comparison of PD-L1 Expression Status between Pure-Solid Versus Part-Solid Lung Adenocarcinomas

**DOI:** 10.3390/biom9090456

**Published:** 2019-09-07

**Authors:** Kenichi Suda, Masaki Shimoji, Shigeki Shimizu, Katsuaki Sato, Masato Chiba, Kenji Tomizawa, Toshiki Takemoto, Junichi Soh, Tetsuya Mitsudomi

**Affiliations:** 1 Division of Thoracic Surgery, Department of Surgery, Kindai University Faculty of Medicine, 377-2 Ohno-Higashi, Osaka-Sayama 589-8511, Japan (M.S.); katsu-sato@med.kindai.ac.jp (K.S.) (M.C.) (K.T.) (T.T.) (J.S.) (T.M.); 2 Department of Thoracic Surgery, Izumi City General Hospital, Izumi 594-1101, Japan; 3 Department of Pathology, Kindai University Faculty of Medicine, Osaka-Sayama 594-0073, Japan

**Keywords:** lung cancer, surgery, ground-glass opacity (GGO), biomarkers

## Abstract

Although lung adenocarcinomas (LADs) with ground-glass opacity (GGO; part-solid tumors) have been shown to differ from those without GGO (pure-solid tumors) in clinicopathological features and prognoses, whether programmed death ligand-1 (PD-L1) protein expression differs between these two tumor types is unclear. This study included 124 patients with clinical T1a–c LAD who received pulmonary resections during 2007–2009. The E1L3N antibody was used to stain for PD-L1 in primary LAD specimens. The specimens were considered PD-L1^+^ if ≥1% of tumor cells showed membrane staining, and were classified as having a high PD-L1^+^ tumor proportion score (TPS) if ≥50% of the tumor cells did so. Among the 124 patients, 45 had part-solid tumors and 79 had pure-solid tumors. These two groups did not significantly differ in terms of clinical factors. However, the rates for PD-L1 positivity (4% vs. 25%, *p* < 0.01) and high PD-L1^+^ TPS (2% vs. 16%, *p* = 0.02) were significantly higher in the pure-solid tumors. The multivariate analyses (logistic regression model) showed that the odds ratios for PD-L1 positivity and high PD-L1^+^ TPS in pure-solid LADs were 5.9 (95% CI; 1.2–29.7) and 8.0 (95% CI; 1.0–63.8), respectively. In conclusion, LADs with GGO were correlated with a lower incidence of PD-L1 expression than pure-solid tumors.

## 1. Introduction

Adenocarcinoma is the most common histological subtypes of non-small cell lung cancer (NSCLC). Although ground-glass opacity (GGO) is a common finding in lung adenocarcinoma (LAD) on chest computed tomography (CT), some LADs do not have GGO components. These are termed pure-solid tumors.

The eighth edition of the TNM classification of lung cancer defined clinical T staging by radiological sizes of the solid components, excluding GGO components [1]. This change from the seventh edition was supported by the fact that solid component sizes correspond better with pathologic invasiveness than do all-tumor sizes [2]. However, even among patients with tumors in the same clinical T categories in the eighth edition, those with LAD tumors that harbored GGO components (i.e., part-solid tumors) reportedly had better prognosis than those with pure-solid tumors, which indicates that pure-solid LADs had higher malignant potential [3,4]. Other studies associated even small GGO components with longer survival, even if solid components predominated [5,6].

Based on such prognostic differences, this study evaluated the differences in the programmed death ligand-1 (PD-L1) expression status (which is often used as a predictive biomarker for immunotherapy response), between these two LAD tumor types.

## 2. Results

### 2.1. PD-L1 Expression Status between Part-Solid and Pure-Solid Tumors

Among the 124 patients, 45 had part-solid tumors and 79 had pure-solid tumors. There were no significant differences observed between these two groups in terms of sex, age, or smoking status. However, the rate of PD-L1 positivity was significantly lower for the part-solid tumors than for the pure-solid tumors (4% vs. 25%, *p* < 0.01; Table 1). In addition, only 2% of the part-solid tumors had high PD-L1 TPS, compared with 16% of the pure-solid tumors (*p* = 0.02).

### 2.2. Multivariate Analysis Using Clinical Factors

Next, a multivariate analysis was performed to evaluate the correlation between the presence of GGO and the PD-L1 expression status, by adjusting for clinical factors that reportedly affect PD-L1 expression. Even after adjusting for clinical factors, the presence of GGO was significantly correlated with a lower incidence of PD-L1 positivity (Table 2). The part-solid tumors were also correlated with a lower incidence of high PD-L1 TPS tumors. The difference was marginally significant (*p* = 0.05; Table 3).

### 2.3. Histological Features and PD-L1 Expression Status

**Figure 1:**
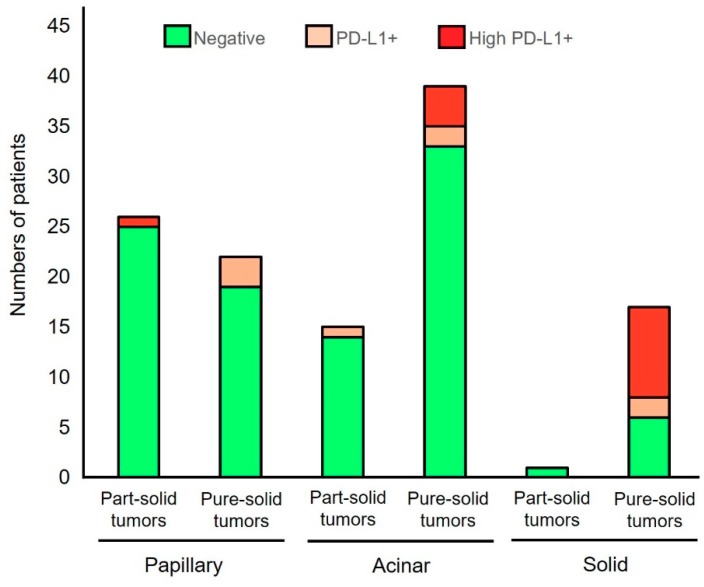
The correlation between PD-L1 positivity and histological subtypes in part-solid LADs and pure-solid LADs. PD-L1+ indicates tumors with PD-L1 membrane staining in 1–49% of tumor cells and high PD-L1+ indicates those with PD-L1 membrane staining in ≥50% of tumor cells.

**Figure 2:**
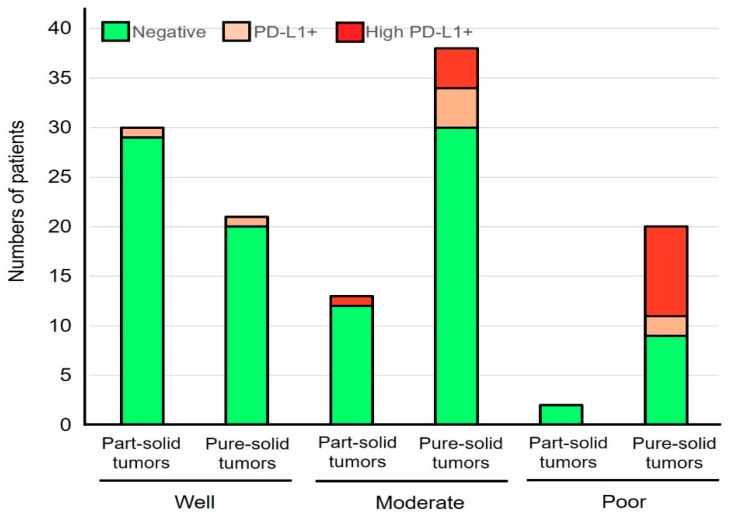
The correlation between PD-L1 positivity and tumor differentiation in part-solid LADs and pure-solid LADs. PD-L1+ indicates tumors with PD-L1 membrane staining in 1–49% of tumor cells and high PD-L1+ indicates those with PD-L1 membrane staining in ≥50% of tumor cells.

The correlation between histological features and PD-L1 expression status in part-solid LAD and pure-solid LAD was also analyzed. In terms of histological subtype, all three tumors with lepidic predominant LAD in part-solid tumors and one invasive mucinous LAD in pure-solid tumors showed negative staining for PD-L1. The rates of PD-L1 positivity in papillary predominant LAD were 3.8% and 13.6% in the part-solid and pure-solid tumors, respectively. The rates of PD-L1 positivity in acinar predominant LAD were 6.7% and 15.4% in the part-solid and pure-solid tumors, respectively. Although there was only one patient with solid-predominant LAD in part-solid tumors, the rates of PD-L1 positivity were 0% and 64.7% in the part-solid and pure-solid LAD with solid predominant histology (Figure 1). A similar result was obtained when the part-solid LADs and pure-solid LADs were classified based on the tumor differentiation (Figure 2).

### 2.4. Prognostic Impact of PD-L1 Expression

**Figure 3:**
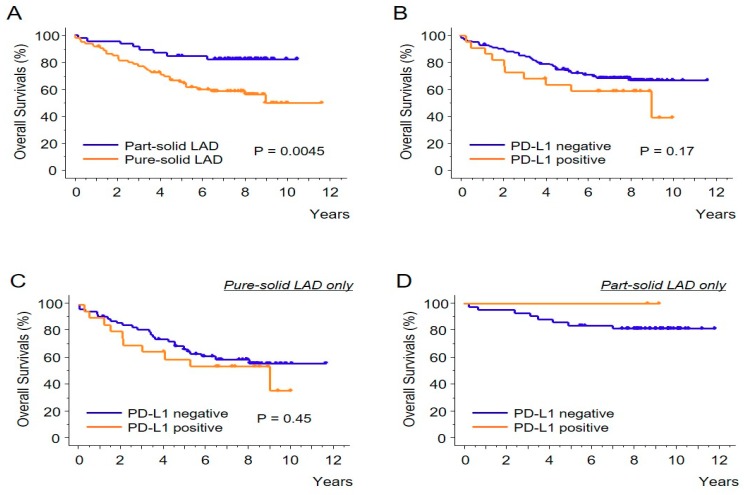
The Kaplan-Meier curves showing the overall survivals. Comparisons of the part-solid LADs versus the pure-solid LADs (**A**) and PD-L1 positive tumors (1% or higher) versus negative tumors (**B**) are shown. The prognostic impact of PD-L1 expression status was also analyzed in pure-solid LADs (**C**) and in part-solid LADs (**D**).

Lastly, the prognostic effect of PD-L1 expression (1% or higher) was evaluated. As expected, the patients with the part-solid LADs had significantly better overall survival compared with the patients with the pure-solid LADs (Figure 3A). In terms of the PD-L1 expression status (Figure 3B), there was a trend of longer survival among the patients with negative PD-L1 expression, although this difference did not reach statistical significance (*p* = 0.17). When the cohort with the pure-solid LADs was evaluated, it was observed that the survival curves of the two groups were almost identical (Figure 3C). As there were only two patients with PD-L1 positivity in the part-solid LAD group, there was no event in the overall survival analysis (Figure 3D).

## 3. Discussion

Recent studies have highlighted the clinical and prognostic differences between LADs with and without GGO [3,4,7,8]. This study observed that these two types of tumors are also different immunologically. Regarding the correlation between the CT appearance and PD-L1 expression, Toyokawa et al. reported that PD-L1 positivity (5% cut-off using SP142 antibody) was significantly associated with the presence of convergence, notching, speculation, and cavitation, and the absence of surrounding GGOs [9]. However, the staining pattern of SP142 antibody is quite different from other PD-L1 antibodies as reported by several comparison studies [10,11]. Therefore, our current results are more substantial than simply confirming the aforementioned study, as the PD-L1 antibody (E1L3N) used in this study has shown a staining pattern similar to that of clinically well-validated antibodies, such as 22C3 [11]. Additionally, clinically meaningful cut-off values (1% and 50%) [12,13] were used in this study. It is of note that this study also suggests that the difference in the PD-L1 status between the part-solid LADs and the pure-solid LADs was not due to the difference in histological subtypes or the difference in tumor differentiation (Figure 1 and Figure 2). In addition, our results may provide important suggestions regarding the prognostic impact of PD-L1 expression which was controversial in previous studies [14]. This study suggests that the prognostic impact of PD-L1 expression is minimal among pure-solid LADs (Figure 3C). However, if the part-solid LADs are combined in the survival analysis, PD-L1 expression may become a poor prognostic factor, since many of the part-solid LADs are negative for the PD-L1 expression (Table 1) and are related to a better prognosis (Figure 3A).

The PD-L1 expression is an important predictor of the efficacy of immunotherapy in patients with metastatic or unresectable NSCLC [15]. Currently, three drugs—pembrolizumab, nivolumab, and atezolizumab—have been approved to treat clinical Stage IV NSCLC. In addition, an anti-PD-L1 antibody drug, durvalumab, has also been approved to treat unresectable Stage III NSCLC after concurrent platinum-based chemotherapy and radiation therapy. Following these successes of immunotherapies in lung cancers, researchers and clinicians are trying these drugs to treat earlier-stage NSCLC in neoadjuvant or adjuvant settings [16]. For example, Forde and colleagues reported, in their pilot study, that two preoperative doses of the PD-1 inhibitor, nivolumab, conferred a major pathological response in 9 of 20 resected tumors (45%) [17]. This study suggests that neoadjuvant or adjuvant immunotherapy is more effective in pure-solid LADs than part-solid tumors, which is meaningful since the pure-solid LADs have significantly higher recurrence risks compared with the part-solid LADs [3,4]. However, the roles of neoadjuvant or adjuvant immunotherapies in earlier-stage NSCLC should be confirmed in clinical trials.

In conclusion, this study found that the incidences of PD-L1^+^ tumors and high PD-L1 TPS lesions were significantly lower in LADs with a part-solid appearance at CT imaging. This pattern may be related to biological differences between these two LAD tumor types.

## 4. Materials and Methods

### 4.1. Patients

This retrospective study included 124 patients with clinical T1a–c LAD (per 8th TNM edition) who received pulmonary resections during 2007–2009. (These patients were also subjects in our previous study [17]). All patients did not receive immunotherapy. This study was approved by the Institutional Review Board of Kindai University Faculty of Medicine (No. 24-253).

### 4.2. Staining

The PD-L1 staining was performed using the E1L3N antibody for tissue microarray samples in our previous study, and was evaluated by a tumor proportion score (TPS). Two cut-offs were used: The PD-L1 status was classified as positive if ≥1% of the tumor cells showed membrane staining; and as high PD-L1 if ≥50% of the tumor cells did so. As the intra-tumor heterogeneity of the PD-L1 expression was anticipated, two cores (with different histological subtypes if applicable) for each specimen were independently analyzed. When the scores of the two cores were different, the higher score for each tumor was adopted. Fifty-seven patients (46.0%) were men, and 43 (40.2%) were smokers. Their median age was 68 years old.

### 4.3. Statistical Analysis

The correlations between the tumor types (part-solid versus pure-solid) and the clinical factors or the PD-L1 expression status were analyzed using the *X*^2^ test. The cut-off value of age was set at the median (68 years old, range 39–83). The univariate and multivariate analyses for factors related to the PD-L1 expression were performed using a logistic regression analysis. The overall survival of the patients in each group was estimated using the Kaplan-Meier method, and the differences were compared using the log-rank test. *p* < 0.05 was considered significant. All analyses were performed using StatView software (v5).

## Figures and Tables

**Table 1:** Characteristics of patients with part-solid or pure-solid lung adenocarcinomas (LAD).

Factors	Part-Solid LAD (*n* = 45)	Pure-Solid LAD (*n* = 79)	*p*-Value
Age			
≤68 year/≥69 year	20 (44%)/25 (56%)	44 (56%)/35 (44%)	0.23
Sex			
Male/Female	20 (44%)/25 (56%)	37 (47%)/42 (53%)	0.80
Smoking status *			
Smokers/Never smokers	13 (33%)/27 (67%)	30 (47%)/34 (53%)	0.15
PD-L1 Expression			
≥1%/<1%	2 (4%)/43 (96%)	20 (25%)/59 (75%)	<0.01
PD-L1 strong expression			
≥50%/<50%	1 (2%)/44 (98%)	13 (16%)/66 (84%)	0.02

* Data not available for 20 patients.

**Table 2:** Univariate and multivariate analyses for factors related to programmed death ligand-1 (PD-L1) positivity ≥ 1%.

Factors	Univariate Analysis		*p*-Value	Multivariate Analysis		*p*-Value
	Odds Ratio	95% CI		Odds Ratio	95% CI	
Age						
≤68 year vs. ≥69 year	8.0	(2.2–28.6)	<0.01	7.0	(1.7–28.6)	<0.01
Sex						
Male vs. Female	1.5	(0.6–3.8)	0.38	-	-	-
Smoking status						
Smoker vs. never smoker	4.3	(1.4–13.5)	0.01	4.0	(1.2–13.9)	0.03
CT findings						
Pure-solid LAD * vs. part-solid LAD	7.3	(1.6–32.9)	<0.01	5.9	(1.2–29.7)	0.03

* LAD: lung adenocarcinoma.

**Table 3:** Univariate and multivariate analyses for factors related to high PD-L1^+^ TPS (≥50%).

Factors	Univariate Analysis		*p*-Value	Multivariate Analysis		*p*-Value
	Odds Ratio	95% CI		Odds Ratio	95% CI	
Age						
≤68 year vs. ≥69 year	3.9	(1.0–14.9)	0.04	3.6	(0.9–13.9)	0.06
Sex						
Male vs. Female	1.7	(0.5–5.1)	0.38	-	-	-
Smoking status						
Smoker vs. never smoker	2.3	(0.6–8.8)	0.22	-	-	-
CT findings						
Pure-solid LAD * vs. part-solid LAD	8.7	(1.1–68.7)	0.04	8.0	(1.0–63.8)	0.05

* LAD: lung adenocarcinomas.
